# A highly active non-precious transition metal catalyst for the hydrogenation of carbon dioxide to formates†
†Electronic supplementary information (ESI) available. See DOI: 10.1039/c8sc05230a


**DOI:** 10.1039/c8sc05230a

**Published:** 2019-05-31

**Authors:** Benjamin G. Schieweck, Niklas F. Westhues, Jürgen Klankermayer

**Affiliations:** a Institut für Makromolekulare und Technische Chemie , RWTH Aachen University , Worringerweg 2 , 52074 Aachen , Germany . Email: jklankermayer@itmc.rwth-aachen.de

## Abstract

Herein a highly active non-precious transition metal catalyst system for homogeneous hydrogenation of carbon dioxide to formate is presented. The application of selected nickel(ii) salts in combination with tailored multidentate ligands enabled the effective transformation of carbon dioxide with an exceptional TON of up to 4.65 × 10^6^. This unprecedented productivity based on the novel nickel catalyst not only outmatches that of existing systems containing first row transition metals, but also established catalysts based on precious transition metals.

## Abstract 2

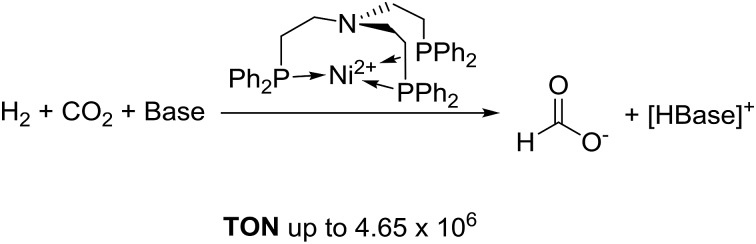
Herein a highly active non-precious transition metal catalyst for the homogeneous hydrogenation of carbon dioxide to formate is presented. Detailed ligand optimisation enabled the development of a nickel-based catalytic system with exceptional productivity.

## 

The utilization of carbon dioxide (CO_2_) as versatile chemical feedstock has fascinated chemists for decades, but only very recently have important developments in molecular catalysis paved the way towards the creation of novel chemical processes using this important resource.^1^–^3^ Especially, the reduction of CO_2_ with molecular hydrogen has been investigated in great detail in the last few years and effective catalysts for the synthesis of formic acid, dimethoxymethane and methanol could be developed.^4^–^9^ In 1935, Farlow and Adkins had already discovered the formation of formates from H_2_ and CO_2_ using a heterogeneous RANEY®-nickel catalyst in the presence of amines.^10^ Later, CO_2_ hydrogenation to formic acid was reported by Inoue *et al.* in 1976 for the first time with a molecular catalyst, and platinum group transition metal complexes modified with mono- and bisphosphine ligands proved to be especially effective.^11^ As the hydrogenation of CO_2_ to formic acid is endergonic, a stabilizing agent, a base or a Lewis basic solvent, is needed to actually yield formates instead of free formic acid.^2^,^12^

Based on this important development, precious metal catalysts achieving TONs of up to several million moles of formate per metal centre could be obtained. In this transformation, molecular iridium and ruthenium catalysts showed by far the highest activity with impressive TONs of up to 3.5 × 10^6^.^13^–^20^ However, important progress could also be achieved with the development of molecular catalysts based on nonprecious metals. In 2003, the Jessop group screened a series of metal salts and found the catalyst NiCl_2_/dcpe (dcpe = Cy_2_PCH_2_CH_2_PCy_2_) with the base DBU (DBU = 1,8-diazabicycloundec-7-ene) as an effective system, resulting in a TON of 4400 after 216 hours at 50 °C.^21^ The Beller group developed Fe and Co catalysts with a tetradentate ligand and in their experiments a TON of up to 1300 (M = Co) could be achieved in the hydrogenation of CO_2_ to dimethyl formamide (DMF).^22^,^23^ An improved iron system, developed by the same group, based on another tetradentate ligand resulted in an even higher TON of 5100 for DMF.^24^ Linehan and co-workers presented a remarkable active *in situ* cobalt system with a bidentate ligand (dmpe = 1,2-bis(dimethylphosphino)ethane) in combination with Verkade's base, resulting in TOFs of up to 74 000 h^–1^ and TONs of up to 9400 at room temperature.^25^ Furthermore, the Linehan group reported of two nickel catalyst systems capable of hydrogenating CO_2_. The first system showed feasibility of CO_2_ hydrogenation in water with a homogeneous nickel catalyst.^26^ Recently, another reported catalyst achieved high TONs for CO_2_ hydrogenation, again relying on the strong Verkade's base. A bimetallic nickel–gallium catalyst was able to perform the reaction at room temperature with TONs of up to 3150 (Fig. 1).^27^

**Fig. 1:**
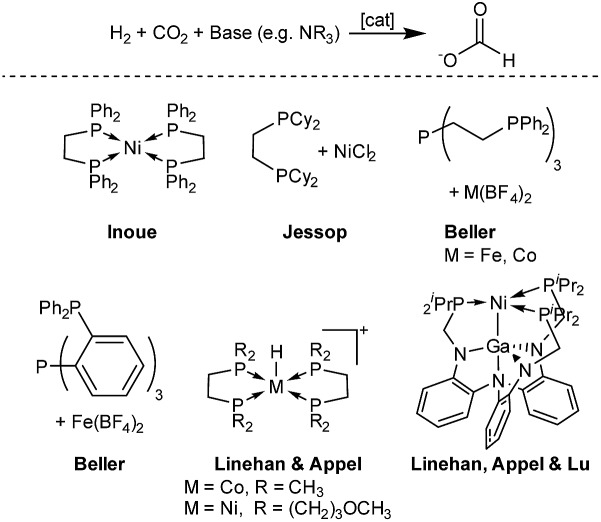
Selected non-precious metal catalysts for the hydrogenation of CO_2_ to formate.^11^,^22^–^27^

In summary, the use of first row transition metals has already been demonstrated, but the achieved TON and TOF values are typically one or two orders of magnitude below those of the best platinum group systems, and highly active and effective systems with non-precious transition metals remain largely elusive.^28^,^29^ Based on the important results achieved with molecular nickel-based catalysts, improved catalyst performance was envisaged with a tailored multidentate ligand structure.

In the first set of CO_2_ hydrogenation experiments a screening of carefully chosen multidentate ligands in combination with Ni(BF_4_)_2_ and DBU as the base was performed (Scheme 1).

**Scheme 1:**
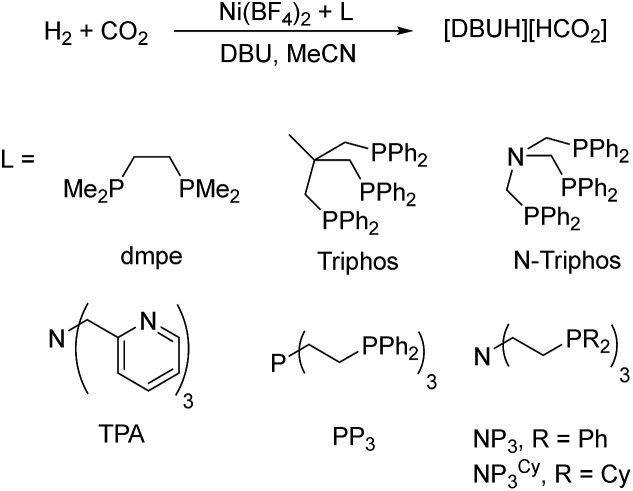
Nickel-catalyzed hydrogenation of CO_2_ with selected multidentate ligands.

Selected results of this initial investigation are summarized in Table 1. In the first experiment with the bidentate phosphine ligand dmpe (1,2-bis(dimethylphosphanyl)ethane) and Ni(BF_4_)_2_ a low TON of 36 could be obtained (Table 1, entry 1). Using the tridentate ligand triphos (1,1,1-tris(diphenylphosphinomethyl)ethane) an increased TON of 79 was achieved and with the related *N*-triphos (*N*,*N*,*N*-tris((diphenylphosphanyl)methyl)amine) a higher TON of 184 was obtained (Table 1, entry 2 and 3). The remarkable difference between these two ligands suggests the importance of an additional coordination site with nickel based molecular systems. However, applying ligands with an additional Lewis basic center and a more flexible ligand backbone showed only a very low catalytic activity. In detail, the reaction with the TPA-based catalyst (TPA = trispyridylmethylamine) resulted in a TON of 14 and with PP_3_ (tris(2-(diphenylphosphino)ethyl)phosphine) a TON of only 41 could be achieved (Table 1, entry 4 and 5).

**Table 1:** Screening of various ligands for the reduction of CO_2_ with Ni^*a*^

Entry	Ligand	TON^*b*^	Yield [%]
1^*c*^	dmpe	36	3.6
2	triphos	79	8
3	*N*-triphos	184	18
4	TPA	14	1.4
5	PP_3_	41	4
6	NP_3_	846	85
7	NP_3_^Cy^	384	38

^*a*^Ni(BF_4_)_2_·6H_2_O (5 μmol), 1 eq. of ligand, *n*_DBU_ = 5 mmol (1000 eq.), *V*_MeCN_ = 2 mL, *T* = 100 °C, *t* = 20 h, *p*(H_2_/CO_2_) [bar/bar] = 60/30 at r. t.

^*b*^TON = *n*_formate_/*n*_cat_ based on integration of the ^1^H-NMR resonances of [DBUH][HCO_2_] with mesitylene as the internal standard.

^*c*^2 eq. of ligand used.

Remarkably, using the tetradentate ligand NP_3_ (NP_3_ = tris(2-(diphenylphosphino)ethyl)amine) showed a high productivity with a TON of 846 (Table 1, entry 6). Using the sterically more strained equivalent NP_3_^Cy^ (NP_3_^Cy^ = tris(2-(dicyclohexylphosphino)ethyl)amine) a decreased turnover number of 384 was obtained (Table 1, entry 7). Based on these results, the NP_3_ ligand showed an excellent performance, and the interplay of the bridgehead nitrogen atom with the nickel center has exceptional importance in the catalyst activity. Recently published results by Mondal *et al.* calculating the energy profile of molecular cobalt and iron catalysts bearing a tetradentate ligand with varying bridgehead atoms corroborate this conclusion.^30^

Consequently, further investigations focused on the application of the versatile NP_3_ ligand in combination with Ni(BF_4_)_2_.

In the first set of experiments, variation of temperature between 80 and 140 °C revealed 120 °C as the optimal reaction temperature. In detail, at 80 °C a TON of 650 was achieved after 20 h, which corresponds to an acid-to-amine-ratio (AAR = *n*_formate_/*n*_DBU_) of approximately 0.33 (Table 2, entry 1). This ratio could be further increased to 0.43 at a temperature of 100 °C (Table 2, entry 2). A nearly full conversion of DBU with a higher AAR of 0.99 was accomplished at 120 °C, resulting in a TON of 1970 (Table 2, entry 3). The reaction at 140 °C led to a significantly increased TON of 1250 after 20 h, but to a maximum AAR of only 0.65 as well due to reduced catalyst stability under these extreme conditions (Table 2, entry 4).

**Table 2:** Hydrogenation of CO_2_ with the developed Ni(BF_4_)_2_/NP_3_ system^*a*^

Entry	*n*(Ni(BF_4_)_2_) [μmol]	*T* [°C]	TON^*b*^	TOF_avg_^*c*^ [h^–1^]	AAR^*d*^
1	5.0	80	650	33	0.33
2	5.0	100	850	43	0.43
3	5.0	120	1970	99	0.99
4	5.0	140	1250	52	0.65
5	0.5	120	20 900	290	1.05
6	0.2	120	54 219	753	1.08
7	0.1	120	93 270	1336	0.93
8	0.04	120	226 630	3148	0.91
9	0.02	120	444 610	6175	0.89
10	0.002	120	4 650 710	64 593	0.93
11^*e*^	5.0	120	0	—	—
12^*f*^	5.0	120	30	0.4	0.02
13^*g*^	—	120	0	—	—
14^*h*^	5.0	120	548	27	0.27
15^*i*^	5.0	120	653	32	0.32
16^*j*^	5.0	120	37	2	0.01

^*a*^Ni(BF_4_)_2_·6H_2_O, 1 eq. of NP_3_, *V*_MeCN_ = 2 mL, *n*_DBU_ = 10 mmol (2000 eq.), *p*(H_2_/CO_2_) [bar/bar] = 60/30 at r. t., entries 1–4, 14–16: *t* = 20 h, entries 5–13: *t* = 72 h.

^*b*^TON = *n*_formate_/*n*_cat_ based on integration of the ^1^H-NMR resonances of [DBUH][HCO_2_] with mesitylene as the internal standard.

^*c*^Given as average TOF, TOF_avg_ = TON/*t* over the whole reaction period.

^*d*^AAR = *n*_formate_/*n*_DBU_.

^*e*^Without DBU.

^*f*^Without NP_3_.

^*g*^ *n*_NP_3__ = 5 μmol.

^*h*^5 eq. of PCy_3_.

^*i*^One additional eq. of NP_3_.

^*j*^1100 eq. of H_2_O (0.1 mL, 5.5 mmol) added.

In the next set of experiments, the catalyst loading was systematically reduced and correlated to the respective TONs (Table 2, entries 5–9). Decreasing the catalyst loading resulted in an increased TON and with 0.002 μmol nickel catalyst an outstanding TON of approximately 4.65 × 10^6^ could be achieved (Table 2, entry 10), surpassing that of established systems based on first row transition metals and even systems based on precious metals.

Control experiments revealed the tailored combination of Ni(BF_4_)_2_, NP_3_ ligand and DBU as the basic requirement for the exceptionally high activity (Table 2, entries 11–13). Recently, Zall *et al.* observed the coordination of the DBU base to the metal center as a crucial factor for a high catalytic turnover in the hydrogenation of CO_2_ with copper catalysts and a comparable pathway may thus be present in the established NP_3_/Ni/DBU system.^29^ Thus, to gain further insight into the complex reaction mechanism, experiments with a defined organometallic compound were performed. Using the molecular nickel(0) complex NP_3_Ni^31^ comparable results and a TON of 2180 could be obtained, thus corroborating the formation of a molecular species during the *in situ* procedure. Furthermore, a mercury-poisoning test showed no inhibition of the catalytic activity. Additionally, an excess of phosphine ligands such as tricyclohexylphosphine (PCy_3_) to a 1 : 1 mixture of Ni^2+^/NP_3_ resulted in a highly decreased TON and a low AAR (Table 2, entry 14). Even the addition of just one additional equivalent of NP_3_ results in reduced activity and a low TON/AAR (Table 2, entry 15). High amounts of water hinder the catalytic hydrogenation of CO_2_. The addition of approx. 1000 equivalents of water leads to inhibition of catalysis (Table 2, entry 16) and the formation of a precipitate. The exact molecular structure of the catalyst remains currently unknown. However, further spectroscopic experiments and mass analysis showed the formation of multiple compounds possibly active in the CO_2_ hydrogenation (see the ESI† for further explanation).

A sequential reaction set-up of subsequently refilling the autoclave with DBU as well as CO_2_ and molecular hydrogen leads to outstanding synthesis of the respective formate on a multigram scale (Scheme 2).

**Scheme 2:**
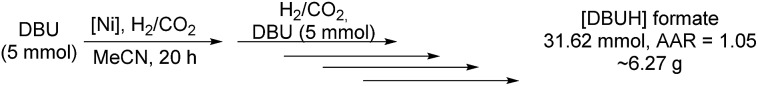
Sequential hydrogenation of CO_2_ by the established nickel catalyst system. 10 mmol of DBU instead of 5 mmol was added in the last reaction step.

Analysis of the reaction mixture after a course of five reactions showed quantitative conversion of the free base resulting in a good TON of 6324 (AAR = 1.05). This productivity denotes an overall yield of roughly 6.30 g (31.62 mmol) of isolable [DBUH] formate and further shows the stability of the nickel catalyst system in this study.

Towards the development of a more effective system and inspired by recent results,^32^–^35^ selected Lewis acidic additives were investigated in combination with the nickel NP_3_ system (Table 3).

**Table 3:** Hydrogenation of CO_2_ with the developed Ni^2+^/NP_3_ system in the presence of selected Lewis acidic additives^*a*^

Entry	Lewis acid (LA)	TON^*b*^	TOF_avg_^*c*^ [h^–1^]	AAR^*d*^
1	—	1545	77	0.31
2	B(C_6_F_5_)_3_	1560	78	0.31
3	Zn(OTf)_2_	1707	85	0.34
4	Al(OTf)_3_	130	7	0.03
5	LiBF_4_	5440	272	1.09
6	LiI	4590	230	0.97
7	LiCl	5140	260	1.08
8	NaBF_4_	400	20	0.09
9^*e*^	LiBF_4_	6260	313	0.63

^*a*^Ni(BF_4_)_2_·6H_2_O (2 μmol) and 1 eq. of NP_3_ used, *n*_LA_ = 200 μmol, *n*_DBU_ = 10 mmol (5000 eq.), *V*_MeCN_ = 2 mL, *p*(H_2_/CO_2_) [bar/bar] = 60/30 at r. t., *T* = 120 °C, *t* = 20 h.

^*b*^TON = *n*_formate_/*n*_cat_ based on integration of the ^1^H-NMR resonances of [DBUH][HCO_2_] with mesitylene as the internal standard.

^*c*^Given as average TOF, TOF_avg_ = TON/*t* over the whole reaction period.

^*d*^AAR = *n*_formate_/*n*_DBU_.

^*e*^ *n*_Ni_ = 1 μmol.

Without any additive, after 20 h a TON of approximately 1500 could be obtained (Table 3, entry 1). Lewis acids such as tris(pentafluorophenyl)borane (B(C_6_F_5_)_3_) or zinc triflate (Zn(OTf)_2_) did not enhance the activity of the established system (Table 3, entries 2 and 3). Aluminium triflate (Al(OTf)_3_) inhibits catalysis and nearly no formate could be observed (Table 3, entry 4). Using lithium tetrafluoroborate (LiBF_4_) a clear enhancement in the catalytic activity was seen achieving a TON of up to 5440 and an AAR of 1.09 (Table 3, entry 5). The tested lithium halides, lithium iodide (LiI) and lithium chloride (LiCl), exhibited a similar behavior in catalysis leading to only slightly decreased values of TONs and AARs (Table 3, entries 5 and 6). Switching from lithium to sodium tetrafluoroborate (NaBF_4_) has a clearly inhibiting effect resulting in only a poor yield of formate in a TON of 400 (Table 3, entry 8). By using LiBF_4_ and additionally lowering the catalyst amount, an AAR of only 0.63 after 20 h with an excellent TON of 6260 could be achieved. Consequently, longer reaction times are required to obtain full conversion of the amine substrate (Table 3, entry 9). To gain further insights into the molecular species formed during catalysis, detailed NMR spectroscopic and mass spectrometric experiments were performed. In detail, ^31^P-NMR-spectroscopy revealed the sole formation of a NP_3_/Ni^2+^ complex by mixing the ligand and Ni(BF_4_)_2_. Analysis after the catalytic experiment revealed a reduction of the initially formed NP_3_Ni(ii) complex, leading to the formation of a major species with a chemical shift of 21 ppm. Confirmation of the defined species NP_3_Ni(0) could be obtained with a further control experiment. Consequently, a mixture of NP_3_/Ni^2+^, DBU and molecular hydrogen (*p* = 3 bar) resulted in a ^31^P-NMR spectrum comparable to that of a *post*-reaction mixture (for details see the ESI†). However, the isolated NP_3_Ni complex did not show evidence of distinct reactivity towards molecular hydrogen and even after prolonged heating at 80 °C, no formation of a hydride species could be observed by NMR spectroscopy. Additional HR ESI-MS measurements confirmed the formation of a NP_3_Ni^2+^ species from the NP_3_/Ni^2+^ mixture during the catalytic experiment (see the ESI†).

## Conclusions

In conclusion, a non-precious transition metal catalyst system could be developed for homogeneous hydrogenation of CO_2_ to formate. The application of the versatile multidentate NP_3_ ligand in combination with selected nickel(ii) salts enabled the effective transformation of CO_2_ in the presence of molecular hydrogen in acetonitrile solution. Under optimized conditions, using 0.002 μmol nickel catalyst, an exceptional TON of approximately 4.65 × 10^6^ was achieved. This unprecedented productivity based on the novel nickel catalyst not only outmatches that of existing systems containing first row transition metals, but also established catalysts based on precious transition metals. Further development and detailed mechanistic investigations on the catalyst system are ongoing in our laboratories.

## Experimental section

The general procedure for homogeneous hydrogenation of carbon dioxide to formate using Ni(BF_4_)_2_·6H_2_O and NP_3_: under an argon atmosphere, a Schlenk tube was charged with a freshly prepared stock solution (*c*_Ni/NP_3__ = 2.5 μmol mL^–1^) of Ni(BF_4_)_2_·6H_2_O and NP_3_. In the case of highly diluted reactions the stock solution was further diluted (see the ESI† for detailed information). DBU was added and the solution was transferred to a stainless steel autoclave equipped with a glass inlet under argon. The autoclave was pressurized with CO_2_ (30 bar) until saturation, followed by hydrogen to a total pressure of 90 bar at room temperature. The reaction mixture was stirred and heated to the respective reaction temperature in an aluminium heating cone. After the reaction period, the autoclave was cooled to room temperature and then carefully vented. Mesitylene was added as the internal standard and the resulting solution was analysed by ^1^H-NMR-spectroscopy. The TONs were found to be reproducible within ΔTON = ±10% in at least two independent runs in selected experiments.

## Conflicts of interest

There are no conflicts to declare.

## Supplementary Material

Supplementary information
