# Supplementary material for: A highly active non-precious transition metal catalyst for the hydrogenation of carbon dioxide to formates
Source: Chem Sci. 2019 May 31;10(26):6519–23. doi: 10.1039/c8sc05230a (PMC6611062; doi:10.1039/c8sc05230a)
Supplement: Supplementary file 1 [file SC-010-C8SC05230A-s001.pdf]

Electronic Supplementary Material (ESI) for Chemical Science.
This journal is © The Royal Society of Chemistry 2019

Supporting Information for the publication

"Highly Active Non-Precious Transition Metal Catalyst for the Hydrogenation of Carbon Dioxide to Formates"

By

Benjamin G. Schieweck, Niklas F. Westhues and Jürgen Klankermayer

Contents

1. General information 2
2. Synthesis of np_3Ni (3) 2
3. Catalytic hydrogenation of carbon dioxide 4
4. Mechanistic Investigations 7

1. General information

Safety advice: High-pressure experiments with compressed gases represent a significant safety risk and must be conducted only following appropriate safety procedures and in conjunction with the use of suitable equipment.

For complex synthesis and catalytic experiments, moisture and oxygen were excluded by working in a glovebox or by using Schlenk techniques. Argon 4.8 (Messer, Germany) was used as inert gas. Glassware was predried in an oven at 100 °C followed by heating under vacuum with a heat gun and refilling with argon at least three times. All solvents were purified by distillation prior to use. Tetrahydrofuran (THF) was degassed by bubbling argon with a frit, dried by passing over activated alumina in steel columns and stored over molecular sieve. Acetonitrile and *n*-Heptane were degassed by distillation under argon and dried over molecular sieves. All reageants were commercially supplied by Aldrich, Fluka and Alfa Aesar and used as received unless stated otherwise. 1,8-Diazabicycloundec-7-ene was distilled under vacuum and dried over molecular sieve. Reaction gases hydrogen (5.0) and carbon dioxide (4.6) were supplied by Linde and PraxAir and used without further purification. *N,N,N*-tris(diphenylphosphinomethyl)amine (N-Triphos)[1], Tris(2-dicyclohexylphosphino)ethyl)amine (NP_3^{Cy})[2] and Tris(2-(diphenylphosphino)ethyl)amine (NP_3)[3] were synthesized by literature procedures.

NMR-spectra were recorded on a Bruker AV400 or a DPX-300 at room temperature. Chemical shifts δ are given in ppm relative to tetramethylsilane (^1H and ^{13}C) and 85% phosphoric acid (^{31}P). First order spin multiplicities are abbreviated as singlet (s), doublet (d), triplet (t), quadruplet (qua), quintet (qui), sextet (sext) and septet (sep). Couplings of higher order or overlapped signals are denoted as multiplet (m), broadened signals as (br. s). First order coupling constants *J* are given in Hz. Assignments are based on attached proton tests (ATP) and 2-D correlation spectroscopy (HSQC, HMQC, HMBC). Mass spectra were recorded on a Finnigan-MAT-95.

2. Synthesis of np_3Ni (3)

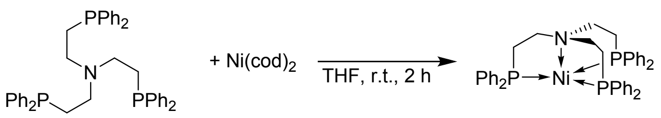

Scheme 1: Synthesis of NP3Ni (**3**) from $Ni(cod)_2$ (**2**) and NP_3 (**1**).

$Ni(cod)_2$ (**2**, 276.1 mg, 1.00 mmol, 1 equiv.) and NP_3 (**1**, 655.1 mg, 1.00 mmol, 1 equiv.) were weighed in, upon addition of THF (10 mL) the solution instantly turned to an intense red. After 2 h of stirring at room temperature, the solvent was removed under reduced pressure and the remaining solid washed with *n*-heptane (3 × 5 mL). After drying in high vacuum the product could be isolated in 88% yield (624.4 mg, 0.88 mmol) as a red powder. Crystalline material could be achieved by slow diffusion of *n*-heptane into a THF solution of the product.

^1H-NMR (400.2 MHz, THF-d_8): δ = 7.10 – 7.19 (m, 12H, C_{Ar}-H), 6.91 – 7.00 (m, 6H, *p*-C_{Ar}-H), 6.78 – 6.88 (m, 12H, C_{Ar}-H), 2.90 (br. s, 6H, P-CH_2), 2.50 (br. s, 6H, N-CH_2) ppm.

$^{13}C\{^1H\}$-NMR (100.26 MHz, THF-d_8): δ = 34.34 (NCH$_2$CH$_2$P), 55.72 (NCH$_2$$CH_2$P), 127.51 ($C_{Ar}$), 128.20 ($C_{Ar}$), 132.69 ($C_{Ar}$) ppm.

$^{31}P\{^1H\}$-NMR (162.0 MHz, THF-d_8): δ = 20.64 (s, 3P) ppm.

MS (FAB-MS) $C_{42}H_{42}NNiP_3$: Calculated: 711.18 m/z. Found: 711.2 (100%) m/z.

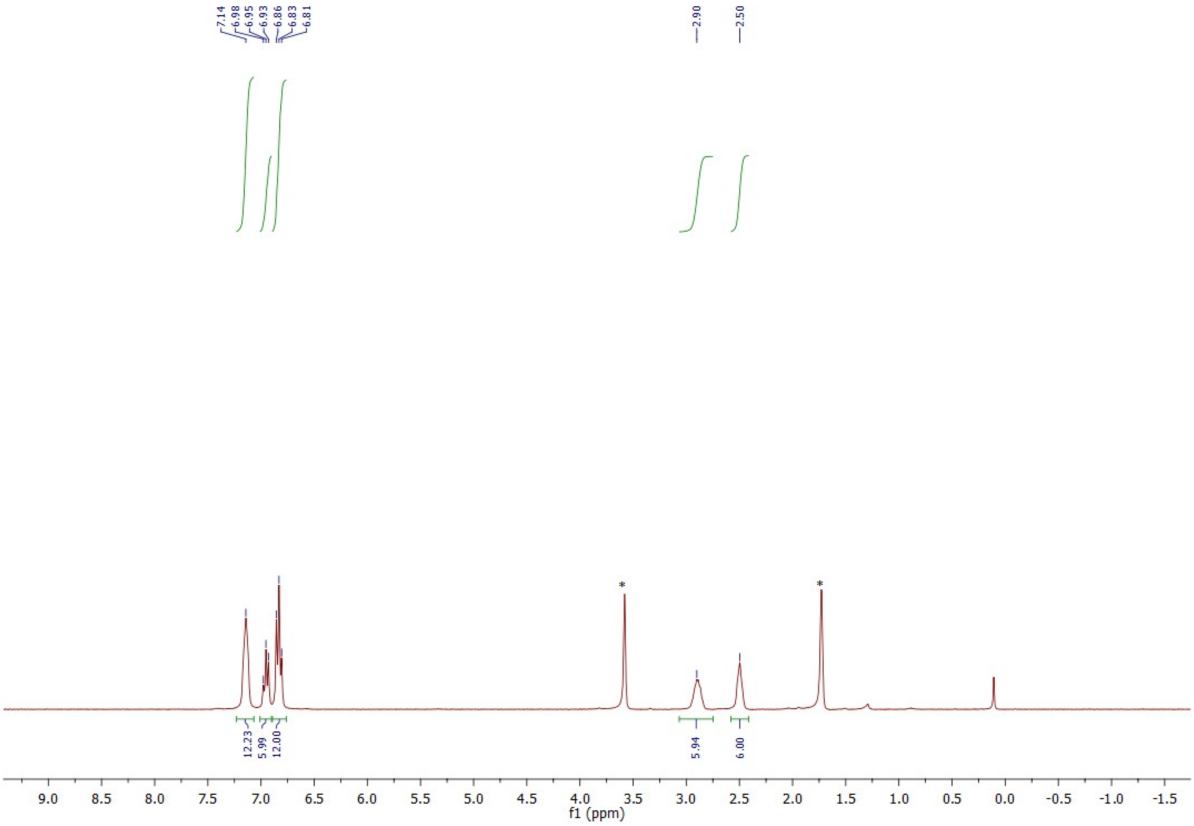

Figure 1: ^{1}H-NMR-spectra of complex **3** (400 MHz, * = THF-d_8).

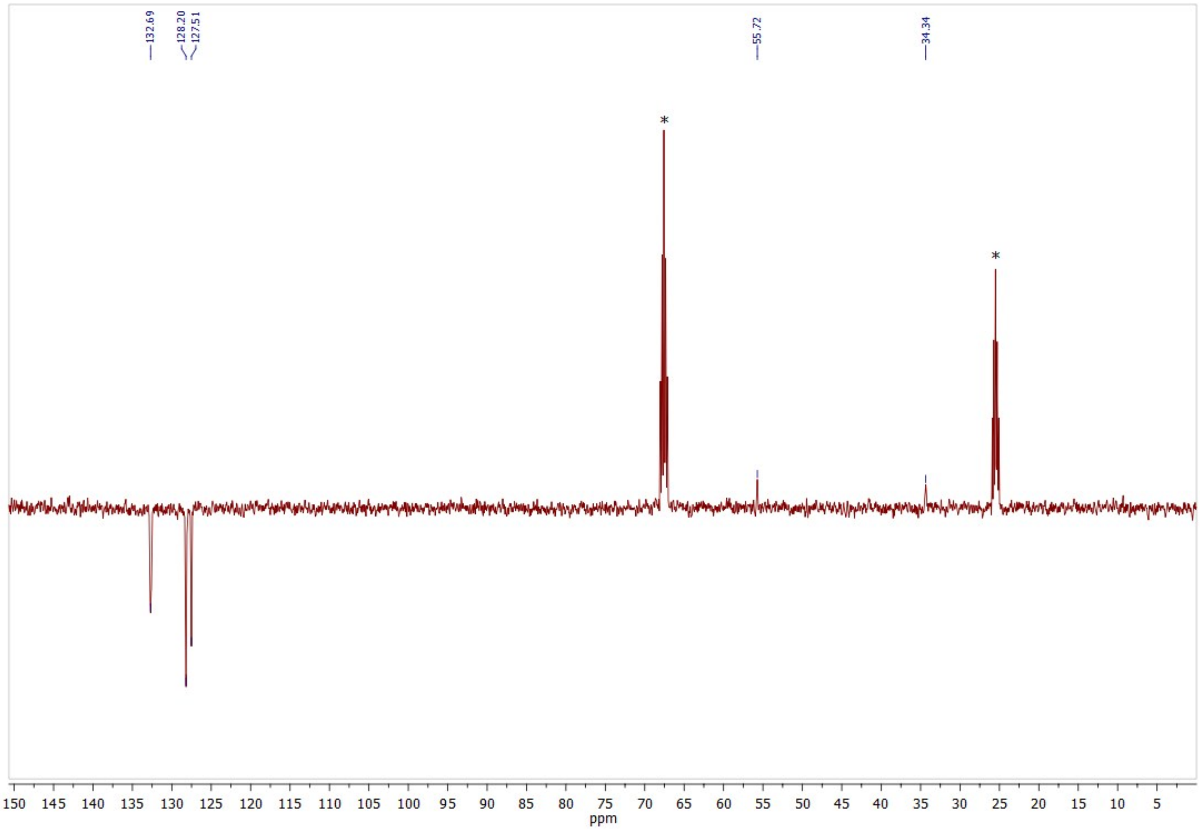

Figure 2: $^{13}C\{^{1}H\}$-APT-NMR-spectra of complex **3** at room temperature (100 MHz, * = THF-d_8).

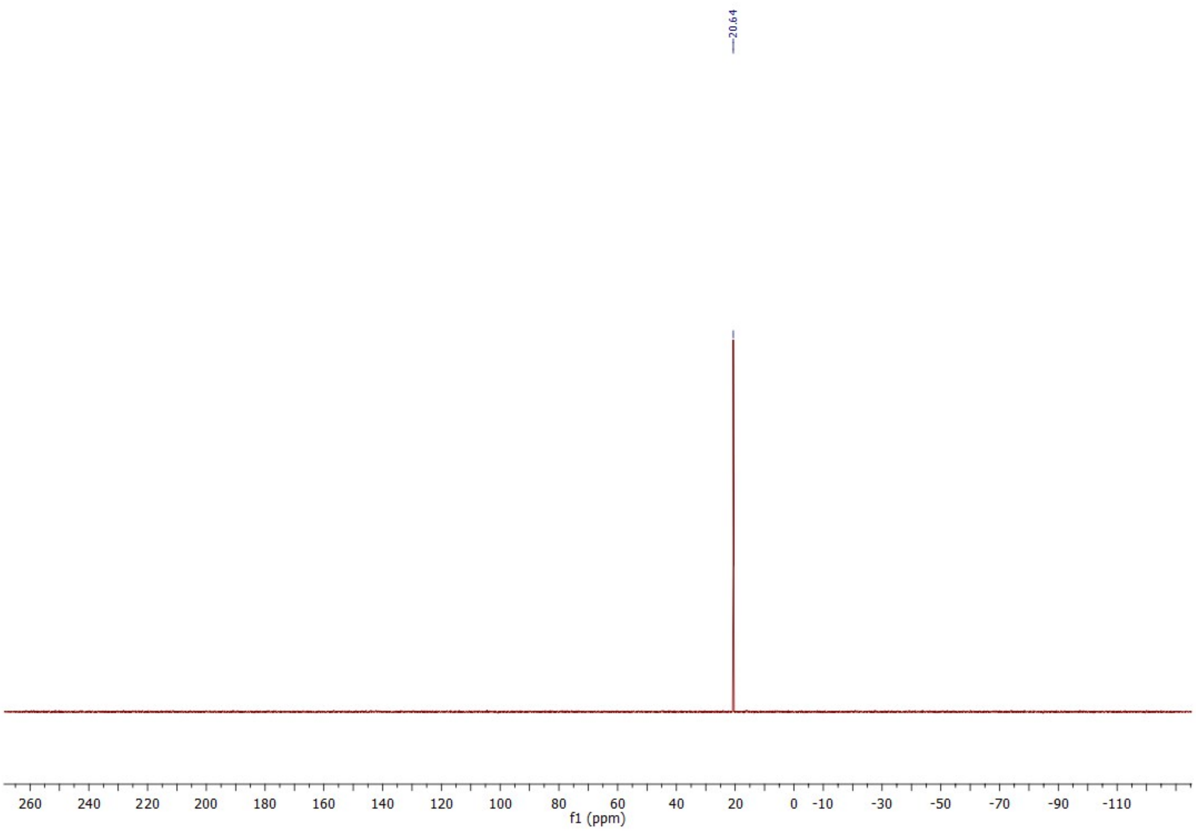

Figure 3: $^{31}P\{^1H\}$-NMR-spectra of complex **3** at room temperature (162 MHz, in THF-d_8).

3. Catalytic hydrogenation of carbon dioxide

a) Exemplary for screening of ligands

In a typical experiment, a Schlenk tube was charged with 2 mL of a freshly prepared stock solution ($c_{Ni/Ligand}$ = 2.5 µmol/mL) of $Ni(BF_4)_2 \cdot 6\ H_2O$ (17.0 mg, 50 µmol) and the respective ligand (50 µmol) in acetonitrile (20 mL). DBU (0.75 mL, 5 mmol) was added and the solution transferred to a 10 mL stainless steel autoclave equipped with a glass inlet and a stirring bar under argon via cannula. The autoclave was pressurized with CO_2 (30 bar) until saturation followed by hydrogen to a total pressure of 90 bar at room temperature. The reaction mixture was stirred and heated to 100 °C in an aluminium heating cone. After 20 hours, the autoclave was cooled to room temperature and then carefully vented. Mesitylene was added as an internal standard and the resulting solution analysed by ^1H-NMR-spectroscopy. TONs were found to be reproducible within ΔTON = ±10% in at least two independent runs in selected experiments.

b) Exemplary for a hydrogenation with NP_3 as ligand

In a typical experiment, a Schlenk tube was charged with a freshly prepared stock solution ($c_{Ni/NP3}$ = 2.5 µmol/mL) of $Ni(BF_4)_2 \cdot 6\ H_2O$ (17.0 mg, 50 µmol) and NP_3 (32.7 mg, 50 µmol) in acetonitrile (20 mL). In the cases of diluted reactions (table 2, entries 5 – 10) the stock solution was further diluted to a concentration of $c_{Ni/NP3}$ = 0.2 µmol/mL, for the reaction with only 0.002 µmol (table 2, entry 10) of catalyst a stock solution with a concentration of $c_{Ni/NP3}$ = 0.02 µmol/mL was used.

In the cases of diluted reactions (table 5, entries 7 – 10) additional acetonitrile was added to a total volume of 2 mL. DBU was added and the solution transferred to a 10 mL stainless steel autoclave equipped with a glass inlet and a stirring bar under argon via cannula. The autoclave was pressurized with CO_2 (30 bar) until saturation followed by hydrogen to a total pressure of 90 bar at room temperature. The reaction mixture was stirred and heated to the respective reaction temperature in an aluminium heating cone. After the reaction time, the autoclave was cooled to room temperature and then carefully vented. Mesitylene was added as an internal standard and the resulting solution analysed by ^{1}H-NMR-spectroscopy (Figure 4). TONs were found to be reproducible within ΔTON = ±10% in at least two independent runs in selected experiments.

c) Exemplary for a hydrogenation in the presence of Lewis acidic additives

In a typical experiment, a Schlenk tube was charged with a freshly prepared stock solution ($c_{Ni/NP3}$ = 2.5 µmol/mL) of $Ni(BF_4)_2 \cdot 6\ H_2O$ (17.0 mg, 50 µmol) and NP_3 (32.7 mg, 50 µmol) in acetonitrile (20 mL). The respective Lewis acid (LA) was either weighed into the glass inlet of the autoclave or a freshly prepared stock solution (c_{LA} = 200 µmol/mL) of the Lewis acid (2.0 mmol) in acetonitrile (10 mL) was used. Additional acetonitrile was added to a total volume of 2 mL, DBU was added and the solution transferred to a 10 mL stainless steel autoclave equipped with a glass inlet and a stirring bar under argon via cannula. The autoclave was pressurized with CO_2 (30 bar) until saturation followed by hydrogen to a total pressure of 90 bar at room temperature. The reaction mixture was stirred and heated to the respective reaction temperature in an aluminium heating cone. After the reaction time, the autoclave was cooled to room temperature and then carefully vented. Mesitylene was added as an internal standard and the resulting solution analysed by ^{1}H-NMR-spectroscopy. TONs were found to be reproducible within ΔTON = ±10% in at least two independent runs in selected experiments.

d) Exemplary for the sequential hydrogenation

A Schlenk tube was charged with a freshly prepared stock solution ($c_{Ni/NP3}$ = 2.5 µmol/mL) of $Ni(BF_4)_2 \cdot 6\ H_2O$ (17.0 mg, 50 µmol) and NP_3 (32.7 mg, 50 µmol) in acetonitrile (20 mL). DBU (0.75 mL, 5 mmol) was added and the solution transferred to a 20 mL stainless steel autoclave equipped with a glass inlet and a stirring bar under argon via cannula. The autoclave was pressurized with CO_2 (30 bar) until saturation followed by hydrogen to a total pressure of 90 bar at room temperature. The reaction mixture was stirred and heated to 120 °C in an aluminium heating cone. After 20 hours, the autoclave was cooled to room temperature and then carefully vented over the Schlenk line. An aliquot (0.2 mL) was taken and analysed by ^{1}H-NMR-spectroscopy. Fresh DBU (0.75 mL, 5 mmol) was added and the procedure was repeated. After run 4, 10 mmol (1.5 mL) of DBU were added as well as additional acetonitrile (1 mL).

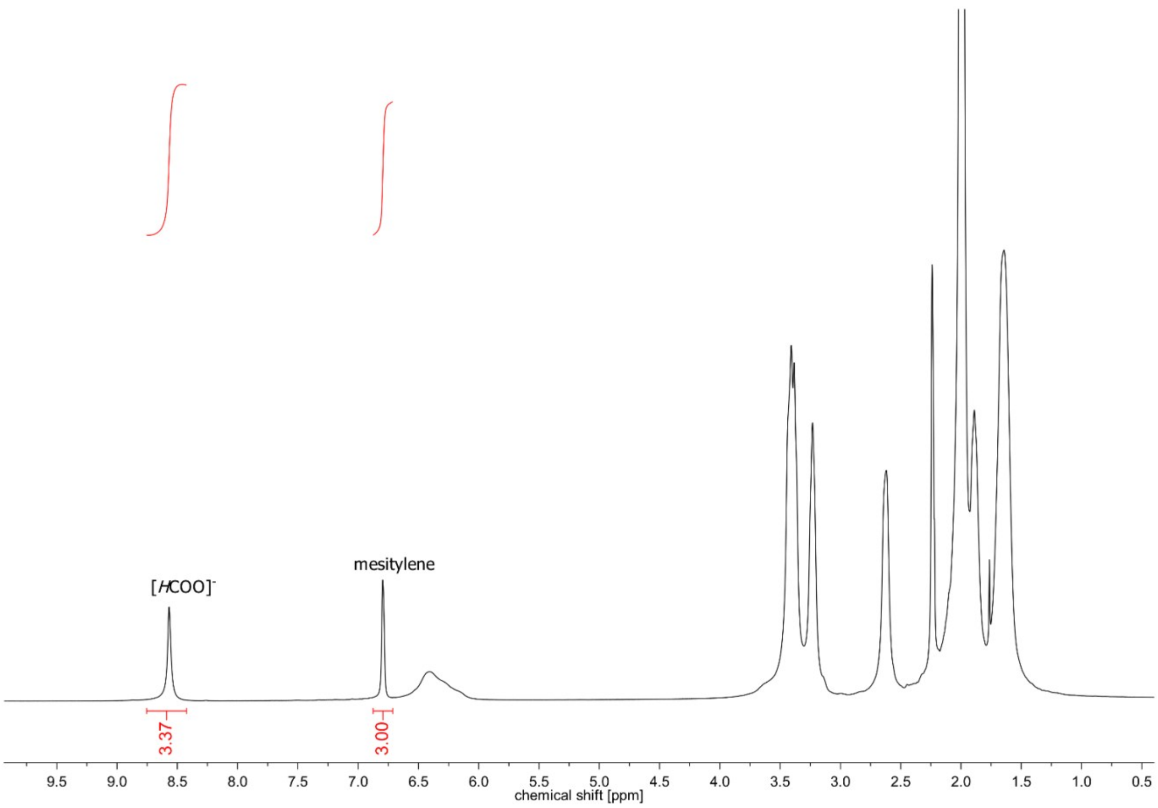

Figure 4: Exemplary ^{1}H-NMR-spectrum for the hydrogenation of CO_2 to [DBUH][HCOO] with Ni^{2+}/NP_3.

4. Mechanistic Investigations

The identification and comprehensive characterisation of the catalytically active species represents a general challenge in molecular catalysis with gaseous reactants. This is especially true in transformations with hydrogen and molecular nickel complexes. Thus, we investigated the presented reaction in detail and added comprehensive information of the catalyst species, acquired with state-of-the art analytic techniques. To gain further insight into possible activation pathways of the catalyst systems, detailed NMR experiments were conducted. Primarily, a mixture of the ligand with $Ni(BF_4)_2$ results in distinctive broad resonances in the $^{31}P\{^1H\}$-NMR spectrum centred at 5 ppm, transforming to several sharp signals after a catalytic experiment (Figure 5).

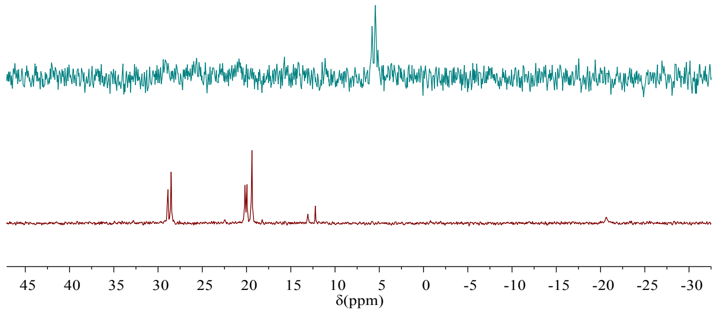

Figure 5: Upper spectrum: $^{31}P\{^1H\}$-NMR of NP_3/Ni^{2+} in THF-*d*$_8$. Lower spectrum: $^{31}P\{^1H\}$-NMR *in-situ* spectrum of a post catalytic mixture in MeCN-*d*$_3$.

At ca. -20 ppm a small broad resonance for non-coordinated NP_3 ligand (**1**) remained in the solution after the catalytic experiment. Major signals can be observed around 20 and 30 ppm and were investigated in detail. The resonance at 20 ppm can be assigned to the defined species NP_3Ni^0, based on a comparable NMR resonance (**3**, 20 ppm). To substantiate this assignment, a solution of NP_3, $Ni(BF_4)_2 \cdot 6\ H_2O$ and DBU was treated with molecular hydrogen (3 bar) in deuterated acetonitrile and lead to an analogous $^{31}P\{^1H\}$-NMR spectrum (Figure 6).

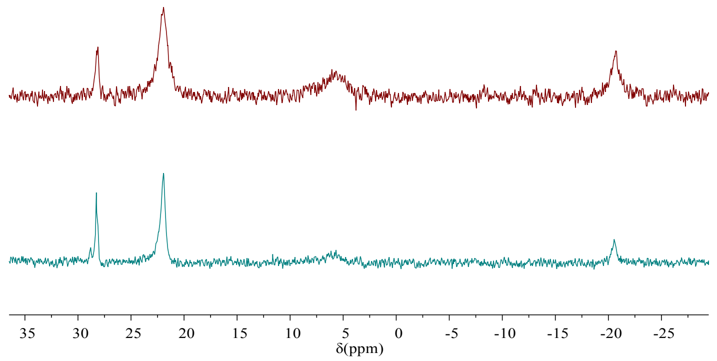

Figure 6: $^{31}P\{^1H\}$-NMR spectra of an *in-situ* treatment of NP_3/Ni^{2+} and base with molecular hydrogen (3 bar) in MeCN-*d*$_3$. Upper spectrum recorded after 20 h at 80 °C, lower spectrum recorded after additional 48 h at 100 °C.

The isolated NP_3Ni (**3**) fragment does not show evidence for distinctive reactivity towards molecular hydrogen. Thus, even after prolonged heating at 80 °C, no formation of a hydride was observed and the compound remained unaltered. Adding DBU as well as molecular hydrogen to **3** did not result in the formation of any hydride species (reaction time up to t = 120 h).

Additional HR-ESI-MS studies prior to catalysis revealed, in accordance to the $^{31}P\{^1H\}$-NMR spectra (see Figure 5), a distinctive formation of a single molecular species with a mass peak of 355.59 m/z, resembling the NP_3/Ni^{2+}-fragment (Figure 7 and Figure 8).

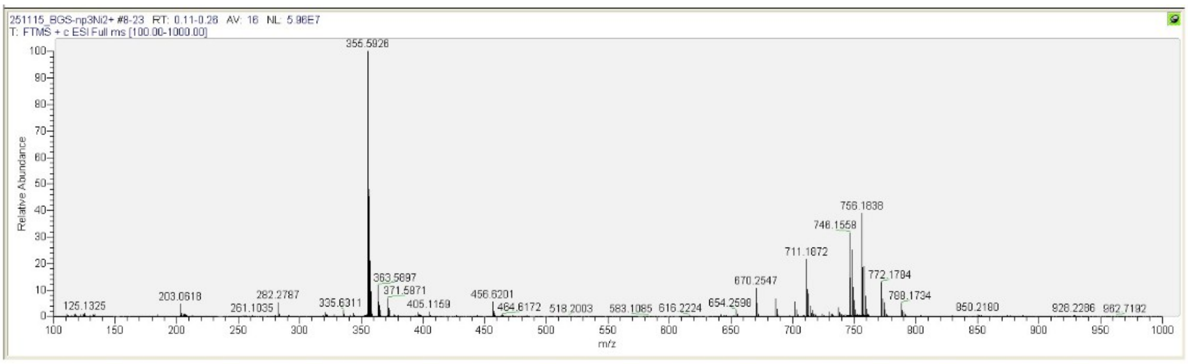

Figure 7: HR-ESI-MS of a mixture of NP_3 and $Ni(BF_4)_2$ in MeCN.

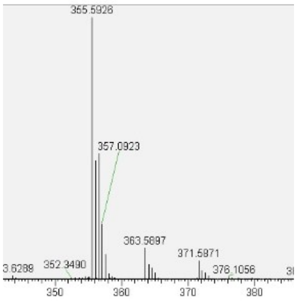

Figure 8: Zoom on the relevant region of the HR-ESI-MS of NP_3/Ni^{2+} in MeCN.

After catalysis HR-ESI-MS measurement were performed, revealing the formation of several compounds (Figure 9) with a distinctive peak at 353.31 m/z. This peak is 2 m/z reduced in comparison to the parent NP_3/Ni^{2+}, hinting towards an oxidation of two arms of the NP_3 ligand (see Scheme 2).

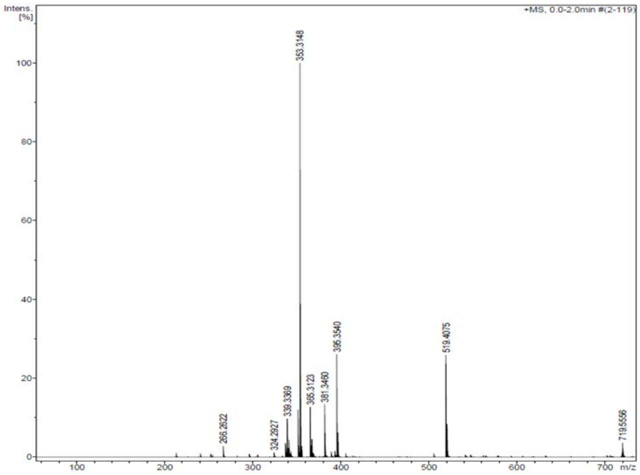

Figure 9: HR-ESI-MS measurement of a post-catalytic mixture.

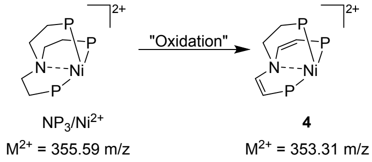

Scheme 2: Possible oxidation of NP_3/Ni^{2+} to complex **4** during catalysis.

Comparable reactivity has been observed by the group of Schneider for their (PNP)Ni-complexes.[4-5] However, it is currently unknown how the oxidation of NP_3 takes place during catalysis and first attempts on the synthesis of complex **4** were unsuccessful.

1. P. W. Miller and A. J. P. White, *J. Organomet. Chem.*, 2010, **695**, 1138-1145.
2. F. Mani and P. Stoppioni, *Inorg. Chim. Acta*, 1976, **16**, 177-179.
3. M. I. García-Seijo, A. Habtemariam, P. del Socorro Murdoch, R. O. Gould and M. E. García-Fernández, *Inorg. Chim. Acta*, 2002, **335**, 52-60.
4. F. Schneck, M. Finger, M. Tromp, S. Schneider, *Chem. Eur. J.* **2016**, *23*, 33-37.
5. F. Schneck, J. Ahrens, M. Finger, A. C. Stückl, C. Würtele, D. Schwarzer, S. Schneider, *Nat. Commun.* **2018**, *9*, 1161.
